# Supplementary material for: Parallel Differentiation and Plastic Adjustment of Leaf Anatomy in Alpine Arabidopsis arenosa Ecotypes
Source: Plants (Basel). 2022 Oct 6;11(19):2626. doi: 10.3390/plants11192626 (PMC9573220; doi:10.3390/plants11192626)
Supplement: Supplementary file 1 [file plants-11-02626-s001.zip › plants-1947341-supplementary.pdf]

Supplemental material

Supplemental figures

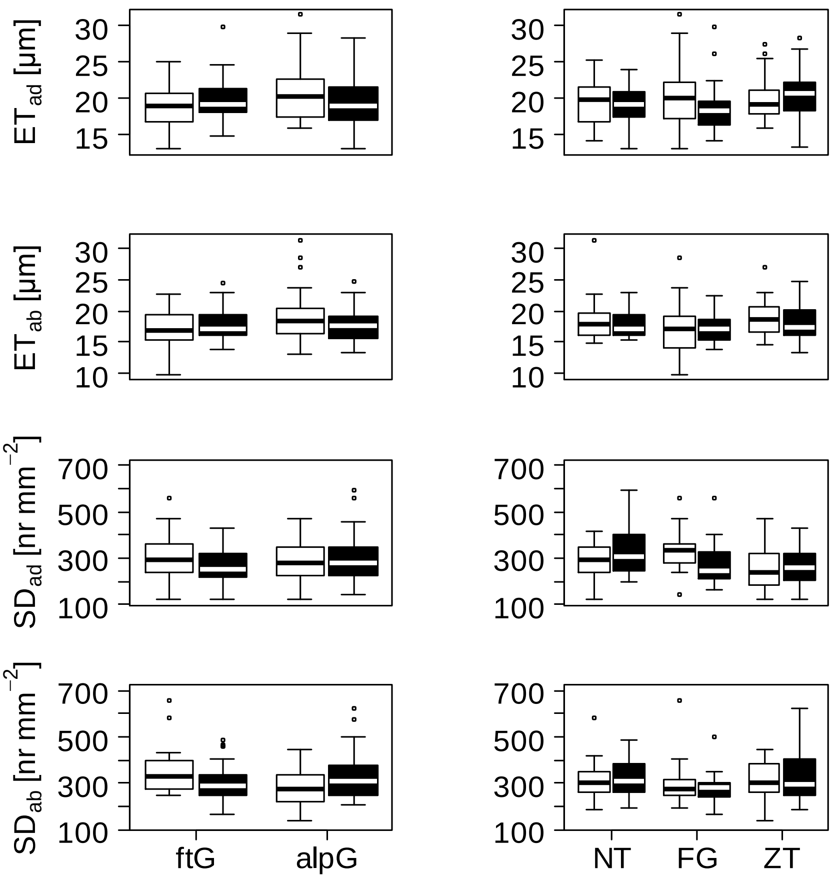

Figure S1. Leaf stomatal density and epidermal thickness in tetraploid *A. arenosa* populations originating from three mountain ranges. Leaf anatomical traits shown are adaxial and abaxial epidermal thickness (ET_{ad} and ET_{ab}), and stomatal density of the adaxial (SD_{ad}) and abaxial (SD_{ab}) leaf surfaces. White boxplots denote alpine populations and black ones show foothill populations grown in a foothill (ftG) and an alpine (alpG) common garden. Box plots show medians and the 25th and 75th percentiles and dots outside 1.5 × interquartile ranges represent outliers. Data of all tetraploid populations per ecotype were pooled, and panels in the left row show comparisons between the two different common gardens and panels in the right row show comparisons across mountain ranges, Niedere Tauern (NT), Fagaras mountains (FG) and Tatra mountains (ZT).

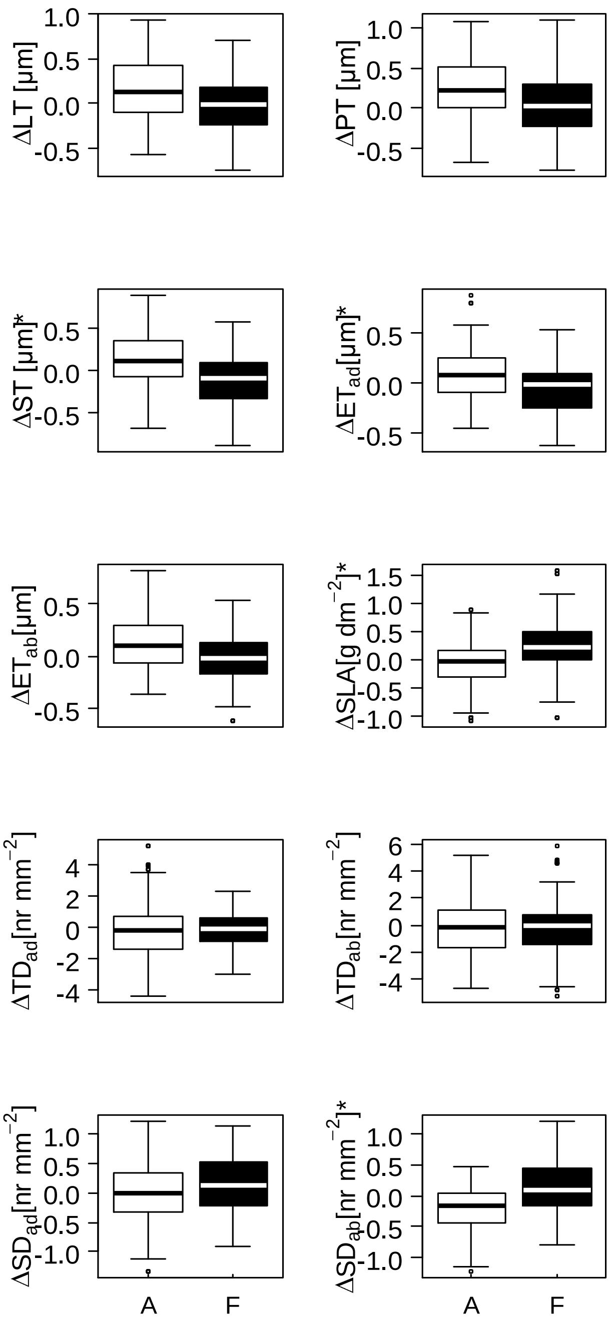

Figure S2. The extent of phenotypic plasticity in leaf traits of tetraploid alpine and foothill *A. arenosa* populations grown in an alpine and foothill common garden. White boxplots denote alpine populations and black ones show foothill populations Differences (Δ) were calculated for each leaf trait by randomly selecting individuals from the same population in each common garden and calculating the log ratio of the trait value observed in the foothill common garden to its value in the alpine common garden. This process was repeated 100 times to ensure randomized pairing. Differences in each leaf trait where then compared using a linear mixed model, with 'ecotype' and 'mountain range' as fixed factor and 'population' as a random factor. Asterisks indicate significant differences in plasticity between ecotypes. Leaf traits analyzed were leaf thickness (LT), palisade parenchyma thickness (PT), spongy parenchyma thickness (ST), epidermis thickness on adaxial (ET_{ad}) and on abaxial (ET_{ab}) leaf surfaces, specific leaf area (SLA), trichome density of adaxial (TD_{ad}) and abaxial (TD_{ad}) and stomatal density of adaxial (SD_{ad}) and abaxial (SD_{ab}) leaf surfaces. Box plots show medians and the 25th and 75th percentiles and dots outside 1.5 × interquartile ranges represent outliers.

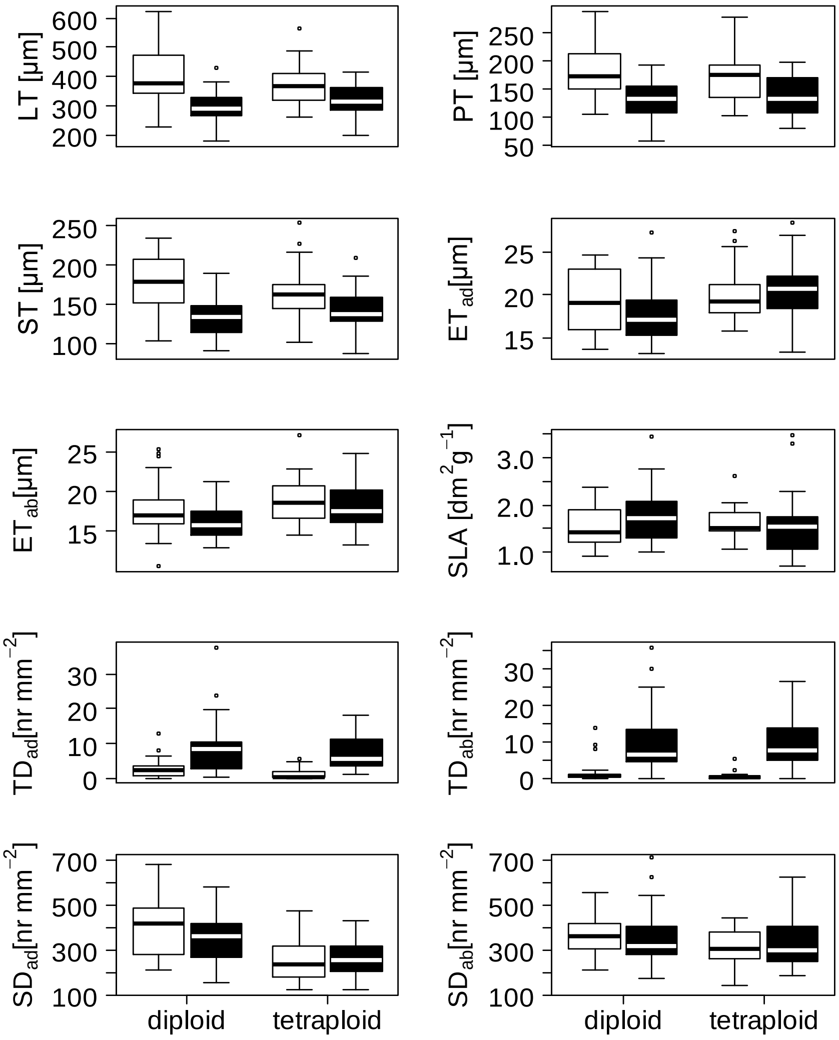

Figure S3. Leaf traits in diploid and tetraploid alpine and foothill *Arabidopsis arenosa* populations. Plants were produced from seeds originating from the Tatra mountains and grown in an alpine and foothill common garden. White boxplots denote alpine populations and black ones show foothill populations. Leaf traits measured were leaf thickness (LT), palisade parenchyma thickness (PT), spongy parenchyma thickness (ST), epidermis thickness on adaxial (ET_{ad}) and on abaxial (ET_{ab}) leaf surfaces, specific leaf area (SLA), trichome density of adaxial (TD_{ad}) and abaxial (TD_{ad}) and stomatal density of adaxial (SD_{ad}) and abaxial (SD_{ab}) leaf surfaces. Box plots show medians and the 25th and 75th percentiles and dots outside 1.5 × interquartile ranges represent outliers.

Supplemental Tables

Table S1. Climatic conditions at the foothill and alpine common gardens used for reciprocal transplantation. Values are daily means ± SD of data recorded between 1st of July and 30th of September 2018 and 2019.

	Climate factor	Foothill habitat (mean ± SD)	Alpine habitat (mean ± SD)
**2018**	**Irradiance (PPFD)**		
	daily sum	65575 ± 34805	87300 ± 37144
	daily maximum	1485 ± 607	2002 ± 594
	**Leaf temperature (°C)**		
	daily maximum	29.9 ± 7.5	17.2 ± 6.8
	daily mean	18.6 ± 4.2	8.5 ± 3.8
	daily minimum	12.7 ± 4.0	3.8 ± 2.8
	**Relative humidity (%)**		
	daily maximum	93.5 ± 11.1	97.2 ± 6.5
	daily mean	77.0 ± 13.3	86.0 ± 13.4
	daily minimum	48.7 ± 19.3	68.0 ± 20.8
**2019**	**Irradiance (PPFD)**		
	daily sum	40373 ± 21888	135914 ± 100608
	daily maximum	1205 ± 550	1912 ± 666
	**Leaf temperature (°C)**		
	daily maximum	28.3 ± 9.5	15.1 ± 9.1
	daily mean	16.3 ± 5.2	7.1 ± 5.2
	daily minimum	10.2 ± 4.3	3.1 ± 3.4
	**Relative humidity (%)**		
	daily maximum	96.5 ± 3.9	98.4 ± 3.3
	daily mean	81.2 ± 11.9	88.4 ± 13.0
	daily minimum	52.8 ± 20.8	72.7 ± 20.6

Table S2. Differences in leaf anatomical traits of tetraploid populations from three mountains ranges grown in a reciprocal transplantation experiment. Type III Analysis of Variance Table obtained by linear mixed models relating ecotype (Ecotype), common garden and mountain range of origin (Region), and the interactions of Ecotype and common garden (Ecotype : Common garden) and of Ecotype and Region (Ecotype : Region) to various leaf anatomical traits. Degrees of freedom were approximated using Satterthwaite's method (see Methods). The nesting of populations within regions was accounted for by introducing a random factor in the model. Cutting direction (angle) was considered by another random factor. Variables were transformed by natural logarithm (log), by square root (sqrt) or cube root (crt) to achieve normal distribution and variance homogeneity.

Factors and interactions	*df*	*Sum of Squares*	*F*	*P*
**Total leaf thickness (LT, log)**				
Ecotype	1	0.728	15.022	**0.008**
Region	2	0.022	0.469	0.646
Common garden	1	0.891	18.397	**<0.001**
Ecotype : Region	2	0.027	0.555	0.601
Ecotype : Common garden	1	0.375	7.732	**0.006**
**Palisade parenchyma thickness (PT, log)**				
Ecotype	1	1.511	18.347	**0.005**
Region	2	0.205	1.244	0.352
Common garden	1	2.791	33.888	**<0.001**
Ecotype : Region	2	0.090	0.549	0.603
Ecotype : Common garden	1	0.196	2.386	0.123
**Spongy parenchyma thickness (ST, sqrt)**				
Ecotype	1	13.585	7.602	**0.033**
Region	2	0.626	0.175	0.844
Common garden	1	1.545	0.865	0.353
Ecotype : Region	2	1.793	0.502	0.629
Ecotype : Common garden	1	47.893	26.803	**<0.001**
**Adaxial epidermis thickness (ET_{ad}, log)**				
Ecotype	1	0.003	0.079	0.788
Region	2	0.120	1.734	0.253
Common garden	1	0.481	13.943	**<0.001**
Ecotype : Region	2	0.028	0.412	0.679
Ecotype : Common garden	1	0.311	9.016	**0.003**
**Abaxial epidermis thickness (ET_{ab}, log)**				
Ecotype	1	0.005	0.111	0.750
Region	2	0.189	2.255	0.186
Common garden	1	0.461	11.016	**<0.001**
Ecotype : Region	2	0.021	0.257	0.781
Ecotype : Common garden	1	0.248	5.924	**0.015**
**Relative proportion of palisade parenchyma (PP_{rel})**				
Ecotype	1	320.17	6.494	**0.044**
Region	2	86.96	0.882	0.462
Common garden	1	2.24	0.045	0.831
Ecotype : Region	2	19.58	0.199	0.825
Ecotype : Common garden	1	1347.66	27.333	**<0.001**

		**Relative proportion of spongy parenchyma (PP_{rel})**		
Ecotype	1	405.03	7.907	**0.005**
Region	2	148.88	1.453	0.235
Common garden	1	154.00	3.007	0.084
Ecotype : Region	2	90.86	0.887	0.413
Ecotype : Common garden	1	1223.78	23.892	**<0.001**
		**Relative proportion of intercellular spaces (IC_{rel}, sqrt)**		
Ecotype	1	0.131	0.290	0.593
Region	2	0.217	0.240	0.788
Common garden	1	0.036	0.079	0.780
Ecotype : Region	2	0.876	0.968	0.389
Ecotype : Common garden	1	0.098	0.216	0.645
		**Specific leaf area (SLA, log)**		
Ecotype	1	0.123	1.529	0.261
Region	2	0.304	1.880	0.231
Common garden	1	0.303	3.747	0.055
Ecotype : Region	2	0.311	1.929	0.224
Ecotype : Common garden	1	0.735	9.108	**0.003**
		**Adaxial trichome density (TD_{ad}, crt)**		
Ecotype	1	9.411	34.291	**0.001**
Region	2	3.951	7.199	**0.029**
Common garden	1	0.226	0.822	0.366
Ecotype : Region	2	1.160	2.112	0.209
Ecotype : Common garden	1	0.063	0.229	0.633
		**Abaxial trichome density (TD_{ab}, crt)**		
Ecotype	1	19.283	39.818	**<0.001**
Region	2	4.049	4.180	0**.074**
Common garden	1	0.065	0.133	0.716
Ecotype : Region	2	2.271	2.345	0.178
Ecotype : Common garden	1	0.573	1.184	0.278
		**Adaxial stomatal density (SD_{ad}, sqrt)**		
Ecotype	1	0.007	0.095	0.759
Region	2	0.660	4.763	**0.010**
Common garden	1	0.027	0.387	0.535
Ecotype : Region	2	0.365	2.636	0.076
Ecotype : Common garden	1	0.082	1.187	0.278
		**Abaxial stomatal density (SD_{ab}, sqrt)**		
Ecotype	1	0.002	0.031	0.866
Region	2	0.072	0.607	0.572
Common garden	1	0.091	1.536	0.218
Ecotype : Region	2	0.052	0.443	0.660
Ecotype : Common garden	1	0.576	9.772	**0.002**

Table S3. Differences in the extent of plasticity for each leaf anatomical trait of tetraploid populations from three mountains ranges grown in a reciprocal transplantation experiment. Type III Analysis of Variance Table obtained by linear mixed models relating ecotype (Ecotype), and mountain range of origin (Region), as well as the interaction of Ecotype and Region (Ecotype : Region) to differences in trait expression in various leaf anatomical traits, i.e. total leaf thickness (LT), thickness of palisade parenchyma (PT), thickness of spongy parenchyma (ST), epidermis thickness on adaxial (ET_{ad}) and on abaxial (ET_{ab}) leaf surfaces, specific leaf area (SLA), trichomes density on adaxial (TD_{ad}) and abaxial (TD_{ab}) leaf surfaces, stomatal density of adaxial (SD_{ad}) and abaxial (SD_{ab}) leaf surfaces. Degrees of freedom were approximated using Satterthwaite's method (see Methods). The nesting of populations within regions was accounted for by introducing a random factor in the model. Differences were calculated by randomly assigning individuals from the same population but different common gardens and using a log ratio of the trait value observed in the foothill common garden to its value in the alpine common garden [$\log(trait_{ftG}/trait_{alpG})$]. The selection process was repeated 100 times.

**Leaf anatomical trait**	*df*	*Sum of Squares*	*F*	*p*
**ΔLT**				
Ecotype	1	0.270	3.453	0.113
Region	2	0.184	1.174	0.371
Ecotype : Region	2	0.205	1.309	0.338
**ΔPT**				
Ecotype	1	0.277	2.330	0.177
Region	2	0.173	0.727	0.522
Ecotype : Region	2	0.240	1.010	0.419
**ΔST**				
Ecotype	1	0.718	8.716	**0.025**
Region	2	0.005	0.031	0.970
Ecotype : Region	2	0.272	1.653	0.268
**ΔET_{ad}**				
Ecotype	1	0.321	7.490	**0.034**
Region	2	0.162	1.889	0.231
Ecotype : Region	2	0.040	0.465	0.649
**ΔET_{ab}**				
Ecotype	1	0.274	5.935	0.051
Region	2	0.053	0.574	0.591
Ecotype : Region	2	0.137	1.484	0.299
**ΔSLA**				
Ecotype	1	1.304	10.803	**0.017**
Region	2	1.210	5.013	0.052
Ecotype : Region	2	0.529	2.192	0.193
**ΔTD_{ad}**				
Ecotype	1	0.429	0.278	0.617
Region	2	11.795	3.823	0.085
Ecotype : Region	2	0.679	0.220	0.809
**ΔTD_{ab}**				
Ecotype	1	0.405	0.138	0.723
Region	2	28.204	4.814	0.057
Ecotype : Region	2	0.928	0.158	0.857
**ΔSD_{ad}**				
Ecotype	1	0.165	0.996	0.357
Region	2	0.431	1.301	0.339
Ecotype : Region	2	0.429	1.297	0.340
**ΔSD_{ab}**				
Ecotype	1	1.286	10.502	**0.018**
Region	2	0.326	1.333	0.332
Ecotype : Region	2	0.077	0.314	0.742

Table S4. Differences in leaf anatomical traits of diploid and tetraploid populations from the Tatra mountains grown in a reciprocal transplantation experiment. Type III Analysis of Variance Table obtained by linear mixed models relating ecotype (Ecotype), common garden and ploidy level (Ploidy), as well as the interaction of ecotype and common garden (Ecotype : Common garden) and the interaction of Ecotype and Ploidy (Ecotype : Ploidy) to various leaf anatomical traits. Degrees of freedom are approximated using Satterthwaite's method (see Methods). The nesting of individuals within populations was accounted for by introducing a random factor in the model. Cutting direction (angle) was considered by another random factor. Variables were transformed by natural logarithm (log), by square root (sqrt) or cube root (crt) to achieve normal distribution and variance homogeneity.

Factors and interactions	*df*	*Sum of Squares*	*F*	*p*
		**Total leaf thickness (LT, log)**		
Ecotype	1	0.274	6.359	0.065
Ploidy	1	0.004	0.091	0.778
Common garden	1	0.423	9.811	**0.002**
Ecotype : Ploidy	1	0.073	1.688	0.262
Ecotype : Common garden	1	0.276	6.405	**0.012**
		**Palisade parenchyma thickness (PT, log)**		
Ecotype	1	0.342	4.639	0.097
Ploidy	1	0.007	0.092	0.776
Common garden	1	0.970	13.170	**<0.001**
Ecotype : Ploidy	1	0.063	0.859	0.405
Ecotype : Common garden	1	0.001	0.007	0.933
		**Spongy parenchyma thickness (ST, sqrt)**		
Ecotype	1	9.803	5.648	0.079
Ploidy	1	0.185	0.106	0.761
Common garden	1	8.202	4.726	**0.031**
Ecotype : Ploidy	1	2.965	1.708	0.262
Ecotype : Common garden	1	57.761	33.280	**<0.001**
		**Adaxial epidermis thickness (ET_{ad}, log)**		
Ecotype	1	0.003	0.089	0.774
Ploidy	1	0.119	1.693	0.260
Common garden	1	0.374	10.672	**0.001**
Ecotype : Ploidy	1	0.030	0.433	0.667
Ecotype : Common garden	1	0.312	8.891	0.003
		**Abaxial epidermis thickness (ET_{ab}, log)**		
Ecotype	1	0.036	0.894	0.399
Ploidy	1	0.089	2.242	0.209
Common garden	1	0.342	8.556	**0.004**
Ecotype : Ploidy	1	0.022	0.565	0.494
Ecotype : Common garden	1	0.151	3.778	0.052
		**Relative proportion of palisade parenchyma (PP_{rel})**		
Ecotype	1	168.56	2.849	0.174
Ploidy	1	64.31	1.087	0.361
Common garden	1	99.62	1.684	0.196
Ecotype : Ploidy	1	29.28	0.495	0.524
Ecotype : Common garden	1	1378.39	23.294	**<0.001**

**Relative proportion of spongy parenchyma (PP_{rel})**				
Ecotype	1	17.01	0.324	0.602
Ploidy	1	27.49	0.524	0.511
Common garden	1	340.44	6.491	**0.012**
Ecotype : Ploidy	1	8.45	0.161	0.710
Ecotype : Common garden	1	1939.26	36.975	**<0.001**
**Relative proportion of intercellular spaces (IC_{rel}, sqrt)**				
Ecotype	1	0.000	0.000	0.999
Ploidy	1	0.130	0.419	0.522
Common garden	1	0.019	0.062	0.805
Ecotype : Ploidy	1	0.079	0.255	0.617
Ecotype : Common garden	1	0.015	0.050	0.825
**Specific leaf area (SLA, log)**				
Ecotype	1	0.107	1.480	0.293
Ploidy	1	0.024	0.335	0.594
Common garden	1	0.468	6.485	**0.013**
Ecotype : Ploidy	1	0.092	1.275	0.323
Ecotype : Common garden	1	1.139	15.768	**<0.001**
**Adaxial trichome density (TD_{ad}, crt)**				
Ecotype	1	0.031	0.131	0.736
Ploidy	1	0.143	0.609	0.479
Common garden	1	3.736	15.911	**<0.001**
Ecotype : Ploidy	1	0.143	0.611	0.479
Ecotype : Common garden	1	0.001	0.006	0.941
**Abaxial trichome density (TD_{ab}, crt)**				
Ecotype	1	0.294	0.875	0.404
Ploidy	1	0.072	0.214	0.668
Common garden	1	6.047	17.989	**<0.001**
Ecotype : Ploidy	1	0.143	0.426	0.549
Ecotype : Common garden	1	0.220	0.654	0.421
**Adaxial stomatal density (SD_{ad}, sqrt)**				
Ecotype	1	0.259	2.285	0.135
Ploidy	1	2.172	19.190	**<0.001**
Common garden	1	0.009	0.079	0.779
Ecotype : Ploidy	1	0.180	1.591	0.211
Ecotype : Common garden	1	0.097	0.856	0.358
**Abaxial stomatal density (SD_{ab}, sqrt)**				
Ecotype	1	0.041	0.613	0.436
Ploidy	1	0.368	5.451	**0.022**
Common garden	1	0.081	1.201	0.276
Ecotype : Ploidy	1	0.033	0.494	0.484
Ecotype : Common garden	1	0.677	10.025	**0.002**

Table S5. Differences in plasticity for each leaf anatomical trait of diploid and tetraploid populations from the Tatra mountains. Type III Analysis of Variance Table obtained by linear mixed models relating ecotype (Ecotype), ploidy level (Ploidy), and their interaction (Ecotype : Ploidy) to differences in leaf anatomical trait expression, i.e. total leaf thickness (LT), palisade parenchyma thickness (PT), spongy parenchyma thickness (ST), epidermis thickness on adaxial (ET_{ad}) and abaxial (ET_{ab}) leaf surfaces, specific leaf area (SLA), trichomes density on adaxial (TD_{ad}) and abaxial (TD_{ab}) leaf surfaces, stomatal density of adaxial (SD_{ad}) and abaxial (SD_{ab}) leaf surfaces. Degrees of freedom were approximated (see Methods). The nesting of individuals into populations within regions was accounted for by introducing a random factor in the model. Differences (Δ) were calculated by randomly selecting individuals from the same population in each common garden and calculating the log ratio of the trait value observed in the foothill common garden to its value in the alpine common garden. The selection process was repeated 100 times.

**Leaf anatomical trait**	*df*	*Sum of Squares*	*F*	*p*
**ΔLT**				
Ecotype	1	0.003	0.041	0.850
Ploidy	1	0.001	0.016	0.905
Ecotype : Ploidy	1	0.004	0.071	0.803
**ΔPT**				
Ecotype	1	0.006	0.058	0.821
Ploidy	1	0.002	0.026	0.880
Ecotype : Ploidy	1	0.005	0.050	0.833
**ΔST**				
Ecotype	1	0.074	1.138	0.346
Ploidy	1	0.084	1.282	0.321
Ecotype : Ploidy	1	0.002	0.038	0.855
**ΔET_{ad}**				
Ecotype	1	0.102	2.286	0.205
Ploidy	1	0.016	0.359	0.582
Ecotype : Ploidy	1	0.049	1.090	0.355
**ΔET_{ab}**				
Ecotype	1	0.000	0.002	0.965
Ploidy	1	0.000	0.008	0.934
Ecotype : Ploidy	1	0.007	0.168	0.703
**ΔSLA**				
Ecotype	1	0.486	3.983	0.117
Ploidy	1	0.226	1.853	0.245
Ecotype : Ploidy	1	0.003	0.029	0.874
**ΔTD_{ad}**				
Ecotype	1	0.098	0.066	0.809
Ploidy	1	0.140	0.095	0.773
Ecotype : Ploidy	1	0.000	0.000	0.993
**ΔTD_{ab}**				
Ecotype	1	3.515	1.658	0.267
Ploidy	1	0.032	0.015	0.907
Ecotype : Ploidy	1	0.124	0.059	0.821
**ΔSD_{ad}**				
Ecotype	1	0.647	1.381	0.305
Ploidy	1	0.073	0.156	0.713
Ecotype : Ploidy	1	0.460	0.981	0.378
**ΔSD_{ab}**				
Ecotype	1	0.254	0.783	0.426
Ploidy	1	0.239	0.736	0.439
Ecotype : Ploidy	1	0.009	0.027	0.878

Table S6. Environmental conditions recorded during the week before sampling: sampling date, day length, minimum and max air temperature (T_{min}, T_{max}).

Common garden	Sampling date	Day/night [h]	T_{min}/ T_{max} [°C]
Foothill common garden, Aigen im Ennstal	16-04-2019	13.5 / 10.5	2 / 9
	19-04-2019	13.5 / 10.5	2 / 15
	24-04-2019	14 / 10	5 / 20
	27-04-2019	14.5 / 9.5	7 / 21
	02-05-2019	14.5 / 9.5	4 / 15
Alpine common garden, Mt. Hohenwart	04-07-2019	17 / 7	6 / 21
	11-07-2019	17 / 7	5 / 11
